# Elastography as a Discriminator Between Fibrotic and Inflammatory Strictures in Crohn’s Disease: A Dead End or Bright Future in Clinical Decision-Making? Critical Review

**DOI:** 10.3390/diagnostics14202299

**Published:** 2024-10-16

**Authors:** Maryla Kuczyńska, Monika Zbroja, Anna Drelich-Zbroja

**Affiliations:** 1 Department of Interventional Radiology and Neuroradiology, Medical University of Lublin, 20-059 Lublin, Poland; 2 Department of Pediatric Radiology, Medical University of Lublin, 20-059 Lublin, Poland

**Keywords:** Crohn, stricture, elastography

## Abstract

Background: Crohn’s disease (CD) is a complex systemic entity, characterized by the progressive and relapsing inflammatory involvement of any part of the gastrointestinal tract. Its clinical pattern may be categorized as penetrating, stricturing or non-penetrating non-stricturing. Methods: In this paper, we performed a database search (Pubmed, MEDLINE, Mendeley) using combinations of the queries “crohn”, “stricture” and “elastography” up to 19 June 2024 to summarize current knowledge regarding the diagnostic utility of ultrasound (US) and magnetic resonance (MR) elastography techniques in the evaluation of stricturing CD by means of an assessment of the transmural intestinal fibrosis. We decided to include papers published since 1 January 2017 for further evaluation (*n* = 24). Results: Despite growing collective and original data regarding numerous applications of mostly ultrasound elastography (quantification of fibrosis, distinguishing inflammatory from predominantly fibrotic strictures, assessment of treatment response, predicting disease progression) constantly emerging, to date, we are still lacking a uniformization in both cut-off values and principles of measurements, i.e., reference tissue in strain elastography (mesenteric fat, abdominal muscles, unaffected bowel segment), units, not to mention subtle differences in technical background of SWE techniques utilized by different vendors. All these factors imply that ultrasound elastography techniques are hardly translatable throughout different medical centers and practitioners, largely depending on the local experience. Conclusions: Nonetheless, the existing medical evidence is promising, especially in terms of possible longitudinal comparative studies (follow-up) of patients in the course of the disease, which seems to be of particular interest in children (lack of radiation, less invasive contrast media) and terminal ileal disease (easily accessible).

## 1. Introduction

Crohn’s disease (CD) is a complex systemic entity, characterized by the progressive and relapsing inflammatory involvement of any part of the gastrointestinal tract. Its clinical pattern may be categorized as penetrating, stricturing or non-penetrating non-stricturing. In stricturing disease, both inflammation and fibrosis contribute to an abnormal narrowing of the intestinal lumen (inflammatory, fibrotic and mixed strictures), with excessive extracellular protein (mainly collagen) deposition. The inflammatory process involves all layers of the intestinal wall and may further progress into transmural fibrotic stenosis, resulting from the uncontrolled and excessive healing process due to the activation of myofibroblasts and a distortion of the mural cytoarchitecture. By definition, a stricture is a localized obstruction of the intestinal lumen by at least 50% compared to the normal segment, accompanied by an apparent upstream dilatation ≥3 cm. In clinical terms, intestinal strictures present as obstructive symptoms, i.e., abdominal cramping, loss of appetite, vomiting, constipation, largely affecting the quality of life and requiring surgical intervention in a substantial percentage of patients [1].

Since to date, no effective anti-fibrotic non-invasive treatment exists, especially for late-stage disease, fibrotic strictures require mechanical interventions, e.g., hemicolectomy or segmental resection, strictureplasty and balloon dilation. On the contrary, inflammatory lesions are easily targeted by means of pharmacological treatment, including anti-inflammatory, immunosuppressive (azathioprine) and biologic (adalimumab) agents. As the inflammatory and fibrostenotic stricturing disease requires a different therapeutic approach, identifying the main component underlying intestinal obstruction by non-invasive means is pivotal in terms of patient’s clinical management [1].

Although conventional cross-sectional imaging (CT and MR enterography) may provide considerable amounts of information regarding the disease burden (extension, activity, intestinal motility), its role is still limited in identifying the leading histopathological cause of strictures. In particular, inflammation and fibrosis concomitantly contribute to strictures in varying proportions in a majority of cases [1]. Herein, diagnostic imaging vendors are in constant search for new technologies that would provide quantitative measures for assessing the level of fibrosis within the affected intestinal loop and thus indicate proper therapeutic strategy. It is stated that sonoelastography meets the above needs, being a promising tool in the evaluation of patients with CD as it can differentiate inflammatory and fibrotic strictures [2].

## 2. Methodology

In this paper, we performed a database search (Pubmed, MEDLINE, Mendeley) using combinations of the queries “crohn”, “stricture” and “elastography” up to 19 June 2024 to summarize current knowledge regarding the diagnostic utility of ultrasound (US) and magnetic resonance (MR) elastography techniques in the evaluation of stricturing CD by means of an assessment of the transmural intestinal fibrosis. A total number of 53 articles were found dating back to 2004; however, due to rapid technological progress (reflected by a sudden growth in the number of publications), we decided to include papers published since 1 January 2017 for further evaluation (*n* = 39). After removal of duplicates and non-full-text publications, a total of 31 records was obtained; 24 were selected for further evaluation after a relevance assessment (1 manuscript was excluded as it concerned methods of liver elastography, 1 for methods of holographic microscopy evaluation, 1 for an ex vivo evaluation of bowel stiffness, and 4 were narrative reviews). These included 12 reviews (2 meta-analysis with systematic reviews, 4 systematic reviews), 11 original scientific papers, and a single case series. Only a single original study concerned MR elastography (Avila 2022) [3]. In addition, we decided to include 2 more recent articles: a meta-analysis and systematic review by Xu et al. (2022) [4] and a systematic review by Lu et al. (2024) [5], as advised by experts.

**Figure 1:**
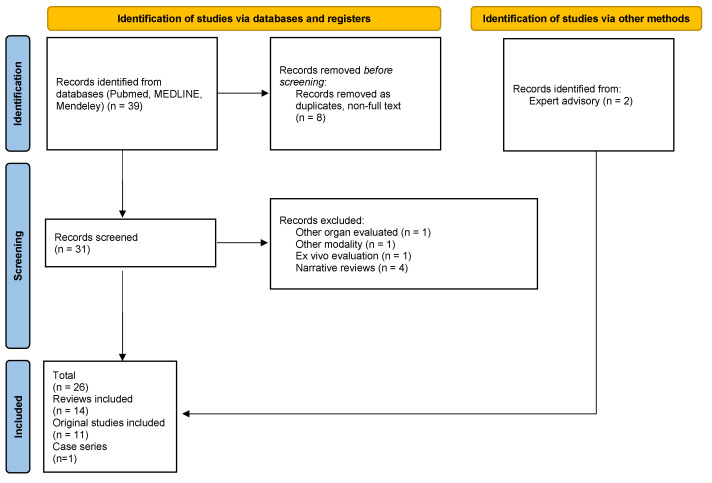
PRISMA flowchart of manuscript selection [own source].

In each case, we obtained the source publications listed in the review series to critically assess the interpretation of the scientific data; whenever applicable, we decided to address the source publication independently to provide a broader description. Original publications not included in the discussed review series were elaborated separately (*n* = 4) [6,7,8,9]. For review series including both human and animal models, only data on humans were further evaluated [10] (Figure 1).

## 3. Elastography as a Measure of Transmural Bowel Fibrosis—Current State of Knowledge

### 3.1. Scientific Evidence from Published Meta-Analyses and Systematic Reviews

In the first meta-analysis on ultrasound elasticity imaging (UEI) in stricturing Crohn’s disease (CD) from 2019 (including scientific papers published before 31 March 2018), Vestito et al. included six studies [10,11,12,13,14,15] with a total of 217 patients with CD, and 231 bowel segments (76 with fibrotic strictures) utilizing either strain (*n* = 4) or shear wave (*n* = 2) elastography (presented as a pooled strain ratio, *n* = 3 or pooled standardized mean strain value, *n* = 3; expressed in meters per second) as a discriminator between inflammatory and fibrotic strictures in patients with CD. Histological or post-surgery specimens served as a reference for the measurement of the degree of fibrosis. Although the strain values were higher in fibrotic lesions, with a standardized mean difference of 0.85 (95% confidence level [CI]: 0 to 1.71) for the strain ratio and 1.0 (95% CI: −0.11 to 2.10) for the mean standardized strain value, the statistical significance was borderline (*p* = 0.05 and *p* = 0.08, respectively). In addition, a high level of heterogeneity was observed between the studies, and nearly all of them (five out of six) were assigned with either high or uncertain risk of bias as evaluated by QUADAS-2 [16].

To no surprise, the results presented in the cited meta-analysis were congruent with the systematic reviews published previously by Pescatori et al. (2018) and Bettenworth et al. (early 2019) [2,17]. Pescatori et al. focused on prospective human studies published between 2008 and 2015, resulting in a total of 129 patients and 154 lesions. Most studies included patients with terminal ileal Crohn’s disease, whereas a single study also allowed for the inclusion of patients with strictures due to other colon pathologies (i.e., adenomas, carcinomas). In six out of seven studies, axial strain elastography was performed, with only a single study utilizing shear wave sonoelastography. Five authors used surgical specimens as a reference, two of them performing US directly on the resected specimens. Authors of the evaluated research used different parameters to assess sonoelastographic data (e.g., absolute strain value, relative strain, semiquantitative scales, ultrasound velocity), aimed at different study endpoints and to some extent presented various approaches for the selection of the examination area, all these reflecting a high level of heterogeneity between the studies. Despite that, all authors managed to find a correlation between the degree of fibrosis and elastography findings, indicating that US elastography can differentiate between inflammatory and fibrotic strictures. Apparently, the wall strain of the fibrotic bowel significantly decreases (both if measured directly, or normalized to external parameters such as unaffected bowel, mesenteric fat and abdominal muscles) [2]. In their systematic review published on behalf of the Stenosis Therapy and Anti-Fibrotic Research (STAR) Consortium, Bettenworth et al. provided a comprehensive definition of CD-related strictures based on an up-to-date research summary on all cross-sectional imaging modalities. One of the main conclusions drawn from the evaluation was a substantial heterogeneity in the definition of stricturing CD used by different authors. Only two studies on US elasticity measurements met the inclusion criteria for the review in terms of separation between inflammatory and fibrotic strictures; both used the strain ratio as a descriptor for stricture characterization and histopathology as a reference [17]. The presented results were contradictory, with Baumgart et al. proving that affected intestinal segments were much less mechanically compliant, with noted strain mean values being significantly higher in unaffected bowel segments (*p* < 0.001), and these findings corresponded well with both increased muscular content and collagen deposition within the intestinal wall on histopathology, as well as with direct tensiometry [14,18]. On the contrary, Serra et al. showed no significant correlations between the degree of inflammation and fibrosis (strain ratio vs. histopathological score of inflammation, *p* = 0.531, fibrosis, *p* = 0.877, clinical/biochemical markers, *p* values between 0.485 for C-reactive protein and up to 0.965 for previous anti-TNF therapy) [13]. 

In the second half of 2021, two more systematic reviews were published, although this time not limited solely to fibrotic strictures but addressing the feasibility of sonoelastography in the assessment of different types of lesions over the course of IBD. 

Ślósarz et al. focused on multiple techniques, including strain (SE) and shear-wave elastography (SWE) and acoustic radiation force impulse (ARFI; otherwise known as point SWE) in IBD, not limiting the study solely to Crohn’s disease. The investigated source data sets were published between 2015 and mid-2021; 12 full-text publications were included in the review (only 1 concerned ulcerative colitis) [19]. Of particular interest were the results presented by Fraquelli et al., who compared the discriminatory value of sonoelastography in the assessment of both inflammatory and fibrotic content of intestinal lesions; the authors found that the strain ratio had an excellent discriminatory ability for diagnosing severe bowel fibrosis, as assessed by the AUROC (strain ratio: 0.917; 95% CI, 0.788–1.000); in addition, a correlation was proved between the wall thickness on conventional US and strain ratios [11,19]. Fufezan et al. established a correlation not only between US strain ratio and the degree of fibrosis but also disease activity markers (but not fecal calprotectin) [19,20]. A whole new chapter started with the era of sonoelastography represented by new quantitative methods—SWE and ARFI. Although Lu et al. (2017) observed higher SWE mean values in patients who required surgical treatment due to stricturing disease compared to those treated conservatively (*p* < 0.01), they failed to find a correlation between SWE values and fibrosis in histopathology samples obtained from 15 surgical patients [14,19]. Chen et al. (2018) struggled to grade fibrosis based on SWE measurements, dividing lesions into mild, moderate and severe according to preset cut-off value ranges in kPa [21]. The authors reached a sensitivity of 69.6% and specificity of 91.7% (AUC 0.822, *p* = 0.002) when applying the cut-off value of 22.55 kPa as a discriminator between mild/moderate and severe fibrosis. In addition, when compared to histology, SWE mean values correlated with the degree of fibrosis, irrespective of inflammation [19,21]. Ding et al. made an attempt to compare real-time or strain elastography with ARFI and p-SWE, using histopathology as a reference [22]. With an optimal cut-off value of shear wave velocity set at 2.73 m/s, only p-SWE reached decent thresholds of sensitivity—75%, specificity—100%, diagnostic accuracy—96%, PPV—100% and NPV—95.5% (AUROC, 0.833; *p* < 0.05) [19,22]. Nonetheless, the major conclusion drawn from the systematic review performed by Ślósarz et al. is that despite promising results indicating sonoelastography as a potential discriminator between the inflammatory and fibrotic strictures, the diversity in technical aspects of measurements between particular elastography techniques (RTE vs. SWE), or even within the same modality but with US systems provided by numerous vendors (measuring slightly different physical properties) hinder direct comparisons and thus translation of the studies into routine clinical practice. This is mainly due to the inability to set the exact cut-off values for significant fibrosis in a uniform manner, using standardized units [19].

Grażyńska et al. evaluated 15 articles from 2011 to 2019 dedicated to the topic of SE and SWE applications in Crohn’s disease only [23]. The authors extracted data from a total 507 patients, 427 evaluated in prospectively designed studies: 2 studies included pediatric patients; 11 assessed strain and 5 shear-wave elastography; and 9 studies used histology as a reference. The majority of the evaluated studies (11 out of 15) overlapped with the analysis performed by Ślósarz et al.; however, data were extracted in a different, more synthetic manner, aiming to compare not only the feasibility of sonoelastography in the characterization of CD strictures but also scientific strategies and methodology (aims/endpoints—detecting fibrosis or inflammation, distinguishing inflammatory and fibrotic strictures, assessing response to anti-TNF treatment, predicting surgery, discriminating between CD and other pathologies; number of investigators; CD activity scales; elastography technique—strain, shear-wave; investigated parameters—qualitative, semi-quantitative, quantitative methods; ROI placement/ reference ROI; source of strain in SE; single vs. multiple measurements at single or multiple time points; units; reference methods—histology/ histopathology or other diagnostic imaging modality) of the source documents. Such an approach enabled them to better depict all the diversity among the studies; not even two of the fifteen studies shared the same methodology [23]. This highlights how important it is to introduce a unified measurement technique (possible guidelines) and parameters (for both strain and shear wave UEI), to prompt further feasibility studies in a clinical setting. Still, only scarce data are available on the only true quantitative modality—SWE. Some authors managed to present a very interesting finding regarding the reproducibility of the elastography measurements; the interobserver agreement in the assessed studies (*n* = 9), expressed either as subjective measures or Kappa values, was reported as moderate to excellence in the majority of cases [23]. Only Havre et al. reported poor agreeability with Kappa value equal to 0.38, in one of the earliest source articles (2014) included in the review [23,24].

The year 2022 brought a new meta-analysis by Xu et al. dedicated to assessing the eligibility of combined ultrasound techniques including B-mode morphometric parameters, contrast-enhanced ultrasound and sonoelastography. In terms of sonoelastography, the authors based their analysis on publications from the years 2014–2019 [11,13,14,22,25,26]. The source data only overlapped with a previous analysis by Vestito et al. from 2019 to some extent, with pooled data including a slightly larger total number of bowel segments of 248. The meta-analysis was focused on evaluating the performance of SR and SV parameters, which both proved to be significantly associated with fibrotic stenosis [SMD = 1.08; 95% CI: 0.47–1.69; *p* = 0.000]. The reported heterogeneity within the selected studies was high [I2 = 70.2% and *p* = 0.003] [3]. 

Some more recent data (from 2022 and 2023) were included in the reviews by Pruijit et al. [27,28,29] and Lu et al. [30,31,32,33,34]—both published in 2024. Apart from Allocca et al. [31], all authors of the new original series utilized shear wave elastography methods in their studies (SWE, p-SWE, ARFI) [28,29,32,33,34]. Differentiating inflammatory from fibrotic stricturing disease was the main focus of the two studies by Allocca [31] and Zhang [33]. Both studies’ authors used a histology assessment of the resected intestinal segments as a reference. Allocca solely evaluated multiple US parameters (including bowel wall thickness and flow signals on color Doppler) along with SE, leading to the conclusion of no significant correlation between sonoelastography and intestinal fibrosis expressed as either collagen or alpha smooth muscle actinin in the full thickness bowel wall or submucosal layer (see Table 1 and Table 2) [31]. On the other hand, Zhang aimed to verify the joint efficacy of SWE and computed tomography enterography (CTE) in differentiating phenotypes of stricturing CD. A positive correlation was observed between the SWE (Emean) and intestinal fibrosis (r = 0.653, *p* = 0.000) with a cut-off value for fibrotic lesions set at 21.30 KPa (AUC: 0.877, sensitivity: 88.90%, specificity: 89.50%, 95% CI: 0.755~0.999, *p* = 0.000). Combining SWE with CTE further improved diagnostic performance and specificity (AUC: 0.918, specificity: 94.70%, 95% CI: 0.806~1.000, *p* = 0.000) [33]. De Cristofaro et al. decided to verify the relationship between the US echopattern of the stricture with the CD clinical behavior and activity. In the analysis, no significant correlation was established between SWE measurements and echopatterns [29]. Wilkens et al., aiming to compare the in vivo preoperative US bowel assessment with the ex vivo examination of the biomechanical properties of the CD strictures on surgical specimens, failed to establish any relevant correlation between US elastography and biomechanical stiffness (see Table 1 and Table 2) [34]. More promising data came from two experiments by Ueno [28] and Matsumoto [32]. Ueno investigated the role of fibrocytes in CD progression to fibrotic patterns and showed that higher numbers of fibrocytes were observed in predominantly fibrotic disease compared to inflammatory strictures, with increased fibrocyte count being strongly correlated with ARFI (see Table 1 and Table 2) [28]. In the study by Matsumoto et al., shear wave elastography proved to be a promising tool in monitoring treatment effectiveness in the fibrotic stricturing CD phenotype [32]. 

All the details on the included meta-analyses, systematic reviews and reviews and their source publications can be found in Table 1 and Table 2, respectively.

### 3.2. Recent Scientific Data—Are We Closer to Definite Cut-Off Values?

Table 3 contains cumulative data from recent publications (2023, 2024) that were not yet published in any of the available review series [6,7,8,9]. Abu-Ata compared sonoelastography (SWE, shear wave speed—SWS) against histological and second-harmonic imaging microscopy (SHIM) in a pediatric population with stricturing CD requiring surgical intervention. Although no direct, relevant correlation was established between the degree of fibrosis and stiffness of the bowel stricture or right quadrant strictures, multiple other interesting correlations were observed, including the one between bowel wall stiffness and smooth muscle hypertrophy (for more details see Table 3) [6]. The second study on a pediatric population by Sidhu et al. aimed to assess combined CEUS and SWE performance in differentiating the CD stricture phenotype. Reportedly, SWE failed to correlate with the degree of fibrosis on histology; however, no exact statistical data were provided by the authors in the manuscript [7]. Chen et al. retrospectively analyzed a cohort of 130 adults with primarily non-stricturing non-penetrating CD to determine the utility of SWE in predicting disease progression to a fibrotic phenotype. In the multivariate analysis, SWE proved to be the only independent variable predicting the disease progression at a cutoff of 12.75 kPa (reverse of the HR) [8]. Kapoor et al. decided to include patients with chronic diarrhea and bowel wall thickening stated on IUS (not limiting themselves to CD or IBD patients) to test SWE in discriminating inflammatory from fibrotic strictures, against CE-CT as a reference standard. According to the authors, SWE not only allowed to differentiate between the two disease phenotypes but was also a useful indicator of the possible underlying etiology of the bowel wall thickening [9].

### 3.3. Magnetic Resonance Elastography

The research strategy retrieved only a single original article—a pilot study by Avila et al. on the performance of magnetic resonance elastography (MRE) in detecting fibrotic lesions and predicting course disease in patients with CD [3]. In this prospective study, the authors included 69 adult patients with a CD diagnosis who underwent contrast-enhanced magnetic resonance enterography and were followed for 450 days afterwards to assess for possible future clinical events (defined as abdominal surgery, hospitalization or consultation at emergency department for abdominal pain or digestive occlusion). All the exams were performed on a 1.5 T scanner with a 30-channel body matrix coil and a 32-channel spine matrix coil for signal reception. One liter of mannitol solution was used as an oral contrast for MRE, and 0.5 mg of glucagon was injected intravenously as intestinal motility inhibitor. Elastography was performed prior to contrast injection. The standard pneumatic active driver system and inversion algorithm (Resoundant, Rochester, MN, USA) were utilized with a frequency of the vibration set at 60 Hz. The passive driver was positioned on the anterior abdomen based on the location of the bowel disease on the morphological sequences. A prototype single-breath-hold multi-slice 2D SE-EPI-based sequence with through-slice motion encoding was used to obtain the magnitude of the complex shear modulus of the affected bowel loops—a measure of stiffness. The region of interest (ROI) on stiffness maps was copied during post-processing from the morphological images at the site of the delineated lesion. Mean stiffness values in kPa were then recorded with 95% confidence intervals (CI). The fibrosis score was assessed visually on a 10-point scale (0 to 9) based on DCE-MRE images by an experienced radiology specialist.

The authors found a significant correlation between the stiffness value measured by MR elastography and the degree of fibrosis based on visual assessment on DCE-MRE (*p* < 0.001). A bowel stiffness ≥ 3.57 kPa predicted the occurrence of clinical events with an area under the curve of 0.82 (95% CI 0.71–0.93; *p* < 0.0001). Unfortunately, no histology verification was introduced in this study to correlate MR findings with degree of fibrosis.

## 4. Conclusions and Future Directions

Despite growing collective and original data regarding numerous applications of mostly ultrasound elastography (quantification of fibrosis, distinguishing inflammatory from predominantly fibrotic strictures, assessment of treatment response, predicting disease progression) constantly emerging, to date, we are still lacking any uniformization in both cut-off values and principles of measurements, i.e., reference tissue in strain elastography (mesenteric fat, abdominal muscles, unaffected bowel segment), units, not to mention subtle differences in technical background of SWE techniques utilized by different vendors. It should be emphasized the considerable heterogeneity of study design—including endpoints, verification method (many examinations did not refer to histopathology) and the parameter evaluated—wave velocity, ARFI, etc. (including different technologies used by different ultrasound manufacturers). All these factors imply that ultrasound elastography techniques are hardly translatable throughout different medical centers, practitioners and ultrasound devices, largely depending on the local experience. Only a few of the cited studies clarified the reproducibility of SWE measurements and stated the interobserver agreement [6,7,11,13,14,18,30,34,43,45]. Sidhu et al., Lu et al., Baumart et al. and Lo Re G et al. introduced no direct interobserver agreement, however, in Fraqueli et al.’s article, interobserver agreement was excellent (between two investigators) with an ICC at 0.90 (95% confidence interval [CI], 0.75–0.96) [7,11,14,18,45]. Mazza et al. evaluated the interobserver agreement in RTE between the same operators in a previous study of their group, which was excellent (intraclass correlation coefficient 0.78; 95% CI 0.42–0.91) [43]. Nonetheless, the existing medical evidence is promising, especially in terms of possible longitudinal, comparative studies (follow-up) of patients in the course of the disease, which seems to be of particular interest in children (lack of radiation, less invasive contrast media) and terminal ileal disease (easily accessible). Also, combining UEI modalities (strain and shear wave elastography) with techniques enabling the assessment of contrast kinetics (CEUS, DCE- or DE-MRE, CE-CT) substantially increased the diagnostic performance of diagnostic imaging in discriminating between CD phenotypes.

Contrary to ultrasound imaging, magnetic resonance elastography seems to have reached a dead end, especially with emerging applications of magnetization transfer imaging to assess fibrotic strictures.

## Figures and Tables

**Table 1:** Evaluated meta-analyses, systematic reviews and reviews and their source publications. Annotations: dark grey—meta analyses, mid-grey—systematic review, light grey—reviews.

Source Publications	Evaluated Review Series								Evaluated Review Series					
	Meta-Analyses and Systematic Reviews								Reviews					
	Lu (2024) [30]	Prujit (2024) [27]	Xu (2022) [4]	Grażyńska (2021) [23]	Ślósarz (2021) [19]	Vestito (2019) [16]	Bettenworth (2019) [17]	Pescatori (2018) [2]	Rimola (2020) [35]	Allocca (2019) [36]	Sousa (2018) [37]	Pita (2018) [38]	Branchi (2017) [39]	Coelho (2017) [40]
Allocca (2023) [31]	X													
Matsumoto (2023) [32]	X													
Zhang (2023) [33]	X													
De Cristofaro (2022) [29]		X												
Ueno (2022) [28]		X												
Wilkens (2022) [34]	X													
Ding (2019) [22]	X	X	X	X	X				X					
Thimm (2019) [41]				X										
Chen (2018) [21]	X	X		X	X				X	X				
Goertz (2018) [42]				X	X									
Mazza (2022) [43]		X												
Orlando (2018) [26]			X	X							X			
Quaia (2018) [44]	X	X		X	X									
Lo Re (2017) [45]				X	X									
Lu (2017) [14]	X	X	X	X	X	X				X	X	X		
Ma (2020) [46]	X													
Orlova (2017) [15]	X		X			X								
Serra (2017) [13]	X	X	X	X	X	X	X			X				
Sconfienza (2016) [47]	X			X	X			X			X			X
Stidham (2016) [12]	X		X			X								
Baumgart (2015) [18]	X			X	X		X	X		X	X	X	X	X
Fraquelli (2015) [11]	X			X	X	X		X	X	X	X	X	X	X
Fufezan (2015) [20]				X	X			X					X	
Dillman (2014) [25]			X					X	X			X	X	X
Havre (2014) [24]				X				X					X	
Bezzio (2013) [48]	X													
Stidham (2013) [49]	X													
Rustemovic (2011) [50]				X										
Stidham (2011) [10]	X					X	X	X			X	X	X	X

**Table 2:** Study details and main conclusions from the source publications used in the evaluated reviews, systematic reviews and meta-analyses presented in Table 1. Abbreviations: %EG—% of enhancement gain; ADC—apparent diffusion coefficient; alpha-SMA—alpha smooth muscle actinin; ARFI—acoustic radiation force impulse; AUC—area under curve; AUROC—area under receiver operating curve; CAL—calprotectin; CD—Crohn’s disease; CEUS—contrast-enhanced ultrasound; CI—confidence interval; CRP—C-reactive protein; CT—computed tomography; CTE—computed tomography elastography; DCE-MRE—dynamic contrast-enhanced magnetic resonance enterography; DE-MRE—delayed-enhancement magnetic resonance enterography; ESR—estimated sedimentation rate; IFX—infliximab; IUS—intestinal ultrasound; MSV—mean strain value; N/A—not applicable; P—prospective; PE—peak enhancement; p-SWE—point shear wave elastography; R—retrospective; SE—strain elastography; SR—strain ratio; SWE—shear wave elastography; SWV—shear wave velocity; TTP—time to peak; UC—ulcerative colitis; UEI—ultrasound elasticity imaging; US—ultrasound; UST—ustekinumab; VTQ—virtual touch quantification (Siemens ARFI application); VT-IQ—virtual touch-IQ (Siemens ARFI application).

Source Publications	Study Design		Number of Patients	Population	Evaluated Intestine Segments	US Elastography		Additional IUS Modalities	Additional Imaging Modalities	Reference (for Intestinal Fibrosis)	US Scanner	Aim of the Study	Main Conclusions
	P/R	US Access				Modality	Assessed Parameters						
Allocca (2023) [31]	P	Transabdominal	17	Adults	affected by stricturing CD (15 terminal ileal, 2 sigmoid)	SE	MSV; color coded maps (strain histogram)	B-mode, Color Doppler US	N/A	Histopathology (surgical specimen)	Aloka Arietta V60 (Hitachi Aloka Medical Ltd., Tokyo, Japan)	To assess the relationsship between various IUS parameters (BWT, BWS, Doppler signals) and SE with histology (inflammation and fibrosis score) findings in CD strictures.	There was no significant correlation between SE (MSV) and bowel fibrosis expressed as: collagen content of either the full bowel wall [ 95%CI: −1.21, 0.07 (*p* = 0.541)] and submucosa layer [95%CI: −0.19, 0.15 (*p* = 0.797)], or total alpha-SMA of either the full bowel wall [95%CI: −0.16, 0.07 (*p* = 0.436)] and sumbmucosal layer [95%CI: −0.25, 0.28 (*p* = 0.925)].
Matsumoto (2023) [32]	R	Transabdominal	21	Not clear	affected by stricturing CD-assessment of treatment response	SWE	SWV	B-mode	colonoscopy, small bowel radiography	N/A (Relative changes in the assessed parameters-shear wave velocity, bowel wall thickness, clinical indices, laboratory indices)	Canon Aplio i900 (Canon Medical Systems Corporation, Otawara, Japan)	Evaluating relative changes in stiffness (SWE, SWV) of the CD strictures before and after treatment with biologics (UST, IFX).	Shear wave elastography might be a useful tool to evaluate treatment response to biologics in patients with stricturing CD. SWV decreased in patients treated with ustekinumab (UST), but not infliximab (IFX) nor those who switched from infliximab to ustekinumab. IFX: 2.24 m/s (1.99–2.63), UST: 2.81 m/s (2.20–4.48), IFX to UST: 3.5 m/s (2.95–4.04). However, no histology reference was provided in this study to quantify the real fibrotic stricture content.
Zhang (2023) [33]	R	Transabdominal	37	>17 years	terminal ileum affected by CD (19 inflammatory and 18 fibrotic segments)	SWE	average value (E mean)	B-mode, Color Doppler US	CTE	Histopathology (surgical specimen)	SuperSonic Imagine AirExplorer (Supersonic Imagine, Aix-en-Provence, France)	To determine the efficacy of both SWE and CTE in differentiating CD phenotypes.	There is a positive correlation between the SWE (Emean) and intestinal fibrosis (Spearman’s r = 0.653, *p* = 0.000). The cut-off value for fibrotic lesions was 21.30 KPa (AUC: 0.877, sensitivity: 88.90%, specificity: 89.50%, 95% CI: 0.755~0.999, *p* = 0.000). Combining the SWE with CTE score (which proved positive correlation with inflammation (Spearman’s r = 0.479, *p* = 0.003) improved diagnostic performance and specificity (AUC: 0.918, specificity: 94.70%, 95% CI: 0.806~1.000, *p* = 0.000) in distnguishing between inflammatory and fibrotic strictures.
De Cristofaro (2022) [29]	P	Transabdominal	40	Not clear	affected/unaffected by CD (disease severity prediction for surgery/ biologics)	SWE	SWV	B-mode, Color Doppler US	N/A	N/A (Comparative study of echopatterns, clinical data, need for steroids, clinical indices and biomarkers and laboratory indices)	No data	To determine the clinical relevance of bowel/ stricture echopattern and its correlation with CD behavior and activity.	No significant correlation was found between SWE values and echopattern groups.
Ueno (2022) [28]	P	Transabdominal	36	Not clear	affected/ unaffected with fibrostenotic CD	SWE-ARFI	SWV	B-mode, Doppler US	N/A	N/A (Comparative study of fibrocytes counts vs. clinical assessment of fibrosis including UEI)	Philips EPIQ 5 (Amsterdam, The Netherlands)	To investigate the role of fibrocytes in the development of fibrosis in CD, and possible correlation with clinical assessment of fibrosis including US.	Fibrocyte numbers were increased in CD patients with stricturing Crohn’s disease compared with patients with an inflammatory phenotype (*p* = 0.0013), with strong correlation between fibrocyte numbers and acoustic radiation force impulse (ARFI) (R = 0.8383, *p* = 0.0127).
Wilkens (2022) [34]	P	Biomechanical parameters: ex vivo examination of the resected specimen, CEUS, UEI: transabdominal in vivo	18	Adults	affected by CD and requiring elective surgical resection	p-SWE-ARFI	SWV	N/A	CEUS, DCE-MRE	Histopathology (surgical specimen, measurement of biomechanical circumferential stricture stiffness-Young’s modulus E)	Siemens Acuson S3000 (Munchen, Germany)	The primary goal was to explore whether parameters obtained by preoperative CEUS and DCE-MRE of strictures associate with biomechanical properties.	There was no correlation between elastography and biomechanical stiffness, E: (ρ = −0.05, *p* = 0.87).
Ding (2019) [22]	P	Transabdominal	25	Adults	affected by stricturing CD	ARFI, SE, p-SWE	SWV	B-mode	CTE, endoscopy	Histopathology (endoscopic biopsies or surgical specimen)	Siemens Acuson S2000	To evaluate the diagnostic performance of SE, ARFI and p-SWE in assessment of the predominant types of intestinal stenosis in CD.	For p-SWE cut-off value of 2.73 m/s (sensitivity of 75% and specificity of 100%) with AUROC of 0.833 [*p* < 0.05] allows to differentiate between a predominantly inflammatory and predominantly fibrotic stenosis.
Thimm (2019) [41]	Case series	Transabdominal	3	Pediatric	affected by CD (acute inflammation, chronic inflammation, post-surgical fibrotic stricture)	SWE	SWV	CEUS, B-mode	MRE	MRE (plus surgical specimen in one case)	Philips EPIQ	To differentiate CD inflammatory and fibrotic strictures by means of SWE and CEUS.	CEUS is most useful in determining the degree of acute inflammation, and quantitative parameters are not as useful in assessing bowel wall fibrosis. Elastography can identify bowel wall fibrosis. Used together, they allow to better differentiate the the degree of fibrosis and inflammation in stricturing CD. However, the elastography value for the patient with active chronic inflammation with stricture formation was comparable to the patient with a surgical fibrotic anastomosis.
Chen (2018) [21]	P	Transabdominal	35	Adults	affected by stricturing CD (ileal/ ileocolonic) requiring surgical resection	SWE	SWV	B-mode	N/A	Histopathology (surgical specimen)	SuperSonic Imagine AirExplorer	To determined if SWE can differentiate between inflammatory and fibrotic strictures of patients with CD.	A cut-off value of 22.55 kPa allows to discriminate between mild/moderate and severe fibrosis with a sensitivity and specificity of 69.9% and 91.7%, respectively [AUC of 0.822, *p* = 0.002]. No significant difference was found between mean SWE values and different grades of inflammation.
Goertz (2018) [42]	P and R	Transabdominal	98 (77 retrospective, 21 prospective)	Adults	affected by CD	ARFI	SWV	B-mode, Doppler US	N/A	Ultrasound signs of activity (bowel wall thickness and Limberg vascularization score), clinical activity and CRP	Siemens Acuson S2000	To evaluate ARFI SWV of the bowel wall in patients with CD with respect to selected clinical and laboratory characteristics (BWT, Limberg score, Harvey-Bradshaw index, CRP).	Retrospectively, the ARFI values correlated with the bowel wall thickness and Limberg vascularization score. Prospectively, there was no correlation between ARFI and bowel wall thickness, Limberg score, clinical activity, and CRP. Therefore, overall ARFI failed to distinguish grades of inflammation wih respect to both US and clinical variables.
Mazza (2022) [43]	P	Transabdominal	40	Adults	ileal/ileoconic CD	SE	SR	B-mode, Doppler US	DE-MRE	DE-MRE	Philips iU22	To evaluate the agreement between RTE and DE-MRE on quantifying CD-related ileal fibrosis.	A significant linear correlation was observed between SR and % of submucosal enhancement gain as compared between MRE images at 70 s and 7 min post gadolinum injection (r = 0.594, *p* < 0.001). Patients with severe fibrosis (SR ≥ 2) had significantly higher submucosal %EG values than patients with low/moderate fibrosis (median values 26.4% vs. 9.5%, *p* < 0.001)
Orlando (2018) [26]	P	Transabdominal	30	Adults	ileal/ileocolonic CD, starting anti-TNF treatment	SE	SR	B-mode, Doppler US	N/A	Histopathology (surgical specimen)	Philips iU22	To assess the ability of UEI to predict the therapeutic outcome for CD patients subjected to anti-TNF treatment.	A significant inverse correlation was observed between the SR at baseline and the thickness variations following anti-TNF therapy [*p* = 0.007]; 27% of patients achieved transmural healing at 14 weeks. The baseline SR was significantly lower in patients with transmural healing [*p* < 0.05].
Quaia (2018) [44]	P	Transabdominal	20	Adults	stricturing terminal ileal CD	SE	SR	CEUS, B-mode	CTE/MRE	Histopathology (endoscopic deep mucosal biopsies or surgical specimen)	Philips iU22	To assess the feasibility of conventional B-mode US, CEUS combined with real-time SE in the differentiation of inflammatory from fibrotic ileal strictures among patients with CD based on visual analysis.	Combination of the conventional B-mode ultrasound, CEUS and SE provides significantly better diagnostic accuracy in distinguishing iflammatory from fibrotic strictures (in terms of specifity, sensitivity, AUROC) compared to implementation of individual techniques [*p* < 0.05].
Lo Re (2017) [45]	P	Transabdominal	35	Adults	41 affected bowel segments, 35 unaffected	SE	SR	B-mode	MRE	MRE (ADC)	Samsung RS80A (Suwon-si, Republic of Korea)	To evaluate and compare the mesenteric and bowel wall changes during CD on SE and MRE	There was a significant correlation between US SE color scale and T2 signal intensity on magnetic resonance images, and between the US-SE color scale and ADC maps.
Lu (2017) [14]	P	Transabdominal	95	Adults	ileal affected by CD (15 requiring surgical resection)	SWE	SWV	CEUS (peak enhancement), B-mode	N/A	Histopathology (surgical specimen)	Siemens Acuson S3000; Philips Epiq 5; Philips IU-22	To correlate SWE of ileal CD in vivo to CEUS peak enhancement and pathology grades of inflammation, fibrosis, and muscular hypertrophy.	There was no significant correlation between SWE and fibrosis scores. However, a moderate correlation between SWE and muscular hypertrophy [r = 0.59, *p* = 0.02] and an inverse relationship between CEUS peak enhancement and SWE velocity measurements [r = −0.061, *p* = 0.03] was observed.
Ma (2020) [46]	P	Transabdominal	20	Adults	affected with CD or UC	SWE	SWV, Young’s modulus	B-mode, Doppler US, CEUS	CTE, colonoscopy	Histopathology (colonoscopic biopsy)	GE LOGIQ E8 (MXR Imaging, San Diego, CA, USA)	To explore the value of multimodal US (B-mode, CEUS, UEI) in the assessment of disease activity and complications in IBD.	The shear wave velocity (3.63 ± 0.86 vs. 2.51 ± 0.66 m/s, *p* = 0.004), Young’s modulus (42.11 ± 14.52 vs. 21.41 ± 8.78 kPa, *p* = 0.001), and TTP value (30.08 ± 6.60 vs. 14.39 ± 3.18 s, *p* < 0.001), PE value (−46.84 ± 7.65 vs. −40.41 ± 5.10 dB, *p* = 0.036), and AUC value (204.38 ± 42.53 vs. 417.95 ± 160.15 dBsec, *p* = 0.001) of inflammatory and fibrotic patient groups were significantly different. Stenosis score was found to be significantly negatively correlated with SWE, Young’s modulus, and TTP [r = −0.593, −0.662, and −0.754 respectively (all *p* < 0.05)]. Stenosis score was found to have a significant positive correlation with PE and AUC [r = 0.450 and 0.643, respectively ( *p* < 0.05)].
Orlova (2017) [15]	R	Transabdominal	24	No data	No data	SE	SR	No data	No data	Histopathology (surgical specimen)	Hitatchi Hi Vision 900	To assess the utility of SE in differentiating between inflammatory and fibrotic CD strictures.	SR values for inflammatory strictures were 1.53 (0.43–3.17), for fibrotic strictures −4.19 (1.57–6.42), statistically significant (Mann-Whitney test, *p* < 0.05).
Serra (2017) [13]	P	Transabdominal	32	Adults	affected by stricturing ileocolonic CD requiring surgical resection	SE	SR	CEUS, B-mode, Color Doppler US	Endoscopy; CTE/MRE	Histopathology (surgical specimen)	Philips iU22	To assess whether real-time SE (SR) could be useful in differentiating fibrotic from inflamed bowel strictures and to evaluate the possible relationship between US techniques and the histology of the stenotic bowel wall in CD patients.	There was no significant correlation between the mean strain ratio and the degree of fibrosis and inflammation (*p* = 0.88 and *p* = 0.53, respectively) on histology.
Sconfienza (2016) [44]	P	Transabdominal	16	Adults	affected by stricturing CD	SE	elastographic map (color scale), semi-quantitative scale	B-mode	MRE	MRE (plus post-surgical histopathology in 2 patients)	Esaote MyLab 70 XvG (Bimedis, East Osceola Parkway Kissimmee, FL, USA)	To test in-vivo real-time SE to differentiate fibrotic from inflammatory terminal ileum strictures in patients with CD, using MRE as a reference standard.	SE of the terminal ileum in CD may differentiate between fibrotic and inflammatory strictures.
Stidham (2016) [12]	P	Transabdominal	28 (9 requiring surgery)	No data	ileal CD (assessment of treatment response)	SWE-ARFI	SWV	N/A	N/A	Relative UEI measurements of SWV at the baseline and at 3 days of methylprednisone therapy	Siemens Acuson S3000	To assess whether assessment of the fibrotic burden in CD patients (by means of ARFI) can distinguish between patients with medical responsive (inflammatory) and non-responsive (predominantly fibrotic) stricturing CD.	Although a tendency was observed that baseline SWVs (with 10% freehand strain to the affected segment) were faster in those undergoing surgical resection, the difference was not statistically significant, 2.02 m/s ± 0.291 versus 1.88 m/s ± 0.254, *p* = 0.128. No significant difference was observed in SWV change between baseline (start of methylprednisone) and day 3 was observed between the surgical and non-surgical groups-ARFI failed to distinguish treatment responsive from non-responsive patients.
Baumgart (2015) [18]	P	Preoperative, intraoperative, postoperative RT-SE	10	Adults	affected by stricturing CD requiring surgery	SE	relative strain measurement; SR	B-mode, Color Doppler US	endoscopy	Histopathology (surgical specimen)	Hitachi Hi Vision	To assess whether real-time SE can be used to detect intestinal fibrosis.	The aggregated SE (RTE) strain mean values were significantly higher in unaffected than in affected bowel segments (mean, 169.0 ± 27.9 vs. 43.0 ± 25.9; *p* < 0.001). There was significant association between SE and collagen depositions.
Fraquelli (2015) [11]	P	Transabdominal	43	Adults	ileal/ileocolonic affected by CD (23 patients witsh strictures requiring surgery, 20 with active, non-stricturing, non-penetrating CD)	SE	SR and color scale (semi quantitative)	B-mode	N/A	Histopathology (in surgical cases)	Philips iU22	To evaluate the feasibility, reliability, and reproducibility of UEI in the assessment of ileal fibrosis in CD patients.	SR of inflammatory CD patients was significantly lower than in patients withe severely fibrotic strictures requiring surgery, and was significantly correlated with the severity of bowel fibrosis at histological analysis. The discriminatory ability for severe bowel fibrosis was high (AUC: 0.917).
Fufezan (2015) [20]	P	Transabdominal	14 (48 bowel segments, 30 ileum, 18 colon)	Pediatric	affected by CD	SE	SR and color scale (pattern)	B-mode, Doppler US	hydrosonography, MRE (6 patients)	colonoscopy, upper GI endoscopy, clinical markers (ESR, CRP, calprotectine), MRE in selected patients	Toshiba Xario V 2.0 (Tokyo, Japan)	To determine whether SE of the bowel wall, in addition to hydrosonography of the colon is a useful tool in the assessment and monitoring of pediatric patients with CD and to propose a SE scoring system for the assessment of CD activity.	There is a significant correlation between SE and SR parameters, and the occurrence of complicating CD (*p* = 0.0430 and *p* = 0.0418). SE and SR were significantly correlated with disease activity markers such as ESR and CRP. However, CAL did not significantly correlate with SR (*p* = 0.1065).
Dillman (2014) [25]	P	Directly on resected specimen (ex vivo)	12 (17 segments, 15 small and 2 large intestine) with known or suspected IBD	Pediatric and Adult	affected by stricturing CD	SWE	SWV	B-mode	N/A	Histopathology (surgical specimen)	Siemens Acuson S3000	To determine if bowel wall fibrosis can be detected by means of SWE (SWV) in fresh ex vivo intestinal specimens.	High–fibrosis score had a significantly greater mean shear wave speed than low–fibrosis score bowel segments (mean ± SD: VTQ, 1.59 ± 0.37 vs. 1.18 ± 0.08 m/s; *p* = 0.004; VT-IQ, 1.87 ± 0.44 vs. 1.50 ± 0.26 m/s; *p* = 0.049). The AUROC for VTQ in differentiating high– versus low–fibrosis score was 0.91 (95% CI, 0.67–0.99; *p*< 0.0001), and for VT-IQ was 0.77 (95% CI, 0.51–0.94; *p* = 0.025). There was no significant correlation between mean shear wave speed and grade of inflammation (VTQ, *p* = 0.22; VT-IQ, *p* = 0.4).
Havre (2014) [24]	P	Directly on resected specimen (ex vivo)	27	Adults	affected by inflammatory and neoplastic (adenocarcinoma) disease	SE	SR and elastorgaphic map (color scale)	B-mode	N/A	Histopathology	Hitachi Hi Vision 900	To investigate whether SE can discriminate between colorectal adenocarcinomas and stenoticCD in newly resected surgical specimens.	The SR of adenocarcinomas (2.51 ± 1.17) was similar CD (3.33 ± 5.21; *p* = 0.537), and both werre significantly higher compared with adenomas (1.31 ± 0.58) (*p* < 0.001). No significant correlation was seen between fibrosis score and SR vs. inflammatory activity.
Bezzio (2013) [48]	R	Transabdominal pre-surgery and ex vivo on the surgical specimen	28	No data	CD strictures od the terminal ileum requiring surgery	SE	SR	B-mode	N/A	Histopathology	Hitachi Logos HiVision C, Esaote MyLab 70 Gold	To evaluate the accuracy of real-time SE in characterising CD strictures, using histology of the resected surgical specimens as a reference.	A significant difference was observed in strain values between fibrotic vs. inflammatory strictures (0.64+/−0.06 vs. 0.91+/−0.08, *p* 0.046) on ex vivo measuremens. In vivo and ex vivo measurements did not differ significantly.
Stidham (2013) [49]	P	Transabdominal	10	No data	obstructive ileal CD (assessment of treatment response to methylprednisone)	SWE-ARFI	SWV	N/A	N/A	Histopathology (surgical specimen)-only 4 patients	Siemens Acuson S3000	To assess whether assessment of the fibrotic burden in CD patients (by means of ARFI) can distinguish between patients with medical responsive (inflammatory) and non-responsive (predominantly fibrotic) stricturing CD-interim analysis.	With 10% freehand strain to the affected segment, baseline SWV showed a tendency towards discrimination between patients requiring surgery within 90 days (2.20 [1.74–2.39] m/s) versus those avoiding surgery (1.81 [1.54–2.09] m/s); statistical significance was not reached, *p* = 0.066. Mean SWV failed to discriminate the two groups at 0% strain. No significant difference in SWV change between baseline and day 3 of methylprednisone was observed between the surgical and non-surgical cohorts.
Rustemovic (2011) [50]	P	Transrectal	83 (30 CD, 25 UC, 28 non-IBD controls)	Adults	affected by CD or UC	SE	SR	N/A	N/A	Histopathology (endoscopy)	Linear echo-endoscope (Pentax FG-38 UX) with the probes of 7, 5–12 MHz (Hitachi EUB 8500)	To assess the role of transrectal ultrasound elastography in distinction between CD and UC.	CD patients with active disease had higher SR than patients in remission (*p* = 0.02). CD patients with active disease had a significantly higher SR compared to UC patients with active disease (*p* = 0.0001).
Stidham (2011) [10]	R	Transabdominal	7	Adults	affected and unaffected bowel segments (total 14)	SE	strain map-used normal bowel as reference; strain value	N/A	CT/MR	Histopathology (surgical specimen) and ex vivo elastometry	Z-1 Zonare Medical Systems (Mountain View, CA, USA)	To assess whether UEI could differentiate inflammatory from fibrotic bowel wall changes in both animal models of colitis and humans with CD.	Bowel wall strain significantly decreases in segments affected by fibrotic stenosis. Mean normalized strain value in stenotic segment was −0.87 ± 0.22 vs. adjacent normal bowel −1.99 ± 0.53 (*p* = 0.0008); the results were confirmed against ex vivo elastometry (Young modulus), with very good (r = −0.81) inverse correlation between pre-operative sonoelastography and ex vivo elastometry.

**Table 3:** Characteristics of the recent publications not included in the investigated review series. Abbreviations: ARFI—acoustic radiation force impulse; AUROC—area under the receiver operating curve; CD—Crohn’s disease; CEUS—contrast-enhanced ultrasound; CE-CT—contrast-enhanced computed tomography; CI—confidence interval; HR—hazard ratio; IUS—intestinal ultrasound MRE—magnetic resonance enterography; N/A—not applicable; P—prospective; R—retrospective; SBWT—small-bowel wall thickness; SWD—shear wave dispersion; SWE—shear wave elastography; SWV—shear wave velocity; UEI—ultrasound elasticity imaging; US—ultrasound.

Source Publications	Study Design		No of Subjects	Population	Evaluated Intestine Segments	US Elastography		Additional IUS Modalities	Additional Imaging Modalities	Reference	US Scanner	Aim of the Study	Main Conclusions
	P/R	US Access				Modality	Assessed parameters						
Abu-Ata (2023) [6]	P	Transabdominal	19 (18 with diagnostic UEI)	Pediatric	stricturing small bowel CD requiring surgery	SWE	SWV	B-mode	N/A	Histopathology, second harmonic imaging microscopy (SHIM) from surgical specimen	Siemens Acuson S3000	To determine the relationship between intestinal wall stiffness on US (SWE) vs. fibrosis and smooth muscle hypertrophy (histology, second harmonic imaging microscopy) in stricturing pediatric CD.	**There was no significant correlations between histological bowel wall fibrosis and any measure of bowel wall or overall right lower quadrant stiffness (all *p*-values > 0.05)** . There were multiple significant positive correlations between histological muscularis mucosa inner layer smooth muscle hypertrophy scores and multiple SWE metrics, including bowel wall stiffness with 10% abdominal strain (r = 0.72, *p* = 0.002) and overall right lower quadrant stiffness with 20% abdominal strain (r = 0.71, *p* = 0.002) a significant negative correlation between entire bowel wall active inflammation and overall right lower quadrant stiffness with 20% abdominal strain (r = −0.60, *p* = 0.009). There was a significant positive correlation between entire bowel wall smooth muscle hypertrophy and bowel wall stiffness with 20% abdominal strain (r = 0.47, *p* = 0.047). The ratio of entire bowel wall histological smooth muscle hypertrophy-to-inflammation was positively correlated with overall right lower quadrant stiffness with 20% abdominal strain (r = 0.67, *p* = 0.002). There were significant negative correlations between histological mucosal active inflammation scores and multiple SWE metrics, including bowel wall stiffness with 10% abdominal strain (r = −0.50, *p* = 0.04) and overall right lower quadrant stiffness with 20% abdominal strain (r = −0.69, *p* = 0.0015).
Sidhu (2023) [7]	P	Transabdominal	25 (11 requiring surgery)	Pediatric	stricturing CD of the terminal ileum	SWE-ARFI	SWV	CEUS, B-mode	MRE/ colonoscopy	Histopathology (tissue biopsy, surgical specimen in 11 cases)	No data	To assess the utility of CEUS in combination with SWE in differentiating fibrotic from inflammatory strictures in children with obstructive CD of the terminal ileum.	**SWE showed no correlation with the degree of fibrosis among patients that required surgical intervention.** Note: Authors provided no statistical data on SWE performance.
Kapoor (2024) [9]	R	Transabdominal	76	No data	patients with chronic diarrhea, abdominal pain with bowel wall thickening determined on IUS (small bowel >3 mm, large bowel >4 mm thickness)	SWE	SWD, Young's modulus (E)	B-mode, Doppler US	CECT	CECT, histopathology (surgical specimen in 18 cases, bioopsy in 23 cases)	No data	To test the role of SWE (SWD, Young's modulus) and IUS in differentiating fibrotic from inflammatory strictures in patients with chronic diarrhea and abominal pain vs. CECT.	**SWE combined with intestinal US is able to differentiate inflammatory from fibrotic bowel wall thickening, and helps to form an etiological diagnosis in patients with chronic diarrhea with increased SBWT** . The sensitivity and specificity of combined SWE with SWD and IUS were 100% and 99% vs. CECT was 78% and 96% respectively with AUROC of 100% and 64%.
Chen (2024) [8]	R	Transabdominal	130	Adults	non-stenotic non-penetrating CD (B1 phenotype)	SWE	average elasticity value (kPa)	B-mode, Doppler US	endoscopy	The definition of stricturing disease was determined based on the obvious symptoms of intestinal obstruction, cross-sectional imaging (including CTE, MRE, and IUS), endoscopy, or disease-related surgery	AirExplorer SuperSonic Imagine	To assess the utility of SWE and other IUS variables in predicting disease progression in CD patients.	**SWE was the only independent predictor of disease behavior progression (HR 1.08, 95%CI 1.03–1.12, *p *= 0.001) on multivariate analysis. A reverse of the HR appeared at the cutoff 12.75 kPa. **
